# αB-Crystallin Alleviates Endotoxin-Induced Retinal Inflammation and Inhibits Microglial Activation and Autophagy

**DOI:** 10.3389/fimmu.2021.641999

**Published:** 2021-03-11

**Authors:** Fangyu Wang, Zhaoxin Jiang, Bingsheng Lou, Fang Duan, Suo Qiu, Zhixing Cheng, Xinqi Ma, Yao Yang, Xiaofeng Lin

**Affiliations:** ^1^State Key Laboratory of Ophthalmology, Zhongshan Ophthalmic Center, Sun Yat-sen University, Guangzhou, China; ^2^Guangdong Provincial People's Hospital, Guangdong Academy of Medical Sciences, Guangzhou, China

**Keywords:** αB-crystallin, microglial activation, autophagy, endotoxin-induced uveitis, ocular inflammatory diseases

## Abstract

αB-Crystallin, a member of the small heat shock protein (sHSP) family, plays an immunomodulatory and neuroprotective role by inhibiting microglial activation in several diseases. However, its effect on endotoxin-induced uveitis (EIU) is unclear. Autophagy may be associated with microglial activation, and αB-crystallin is involved in the regulation of autophagy in some cells. The role of αB-crystallin in microglial autophagy is unknown. This study aimed to explore the role of αB-crystallin on retinal microglial autophagy, microglial activation, and neuroinflammation in both cultured BV2 cells and the EIU mouse model. Our results show that αB-crystallin reduced the release of typical proinflammatory cytokines at both the mRNA and protein level, inhibited microglial activation in morphology, and suppressed the expression of autophagy-related molecules and the number of autophagolysosomes *in vitro*. In the EIU mouse model, αB-crystallin treatment alleviated the release of ocular inflammatory cytokines and the representative signs of inflammation, reduced the apoptosis of ganglion cells, and rescued retinal inflammatory structural and functional damage, as evaluated by optical coherence tomographic and electroretinography. Taken together, these results indicate that αB-crystallin inhibits the activation of microglia and supresses microglial autophagy, ultimately reducing endotoxin-induced neuroinflammation. In conclusion, αB-crystallin provides a novel and promising option for affecting microglial autophagy and alleviating symptoms of ocular inflammatory diseases.

## Introduction

Uveitis, a common ocular inflammatory disease, is a leading cause of blindness worldwide affecting individuals regardless of age, sex, or race (1, 2). Corticosteroids are the main treatment option for noninfectious uveitis (3, 4). In addition to poor response by 30% of patients (5), corticosteroid treatment is also accompanied by inevitable systemic as well as ocular side effects (6, 7). Therefore, there is a need for an efficient and safe intervention for uveitis. Microglia, the primary resident population of innate immune cells in the brain and retina, activates under stress and produces proinflammatory neurotoxic cytokines such as the tumor necrosis factor (TNF) and nitric oxide (NO), leading to a cascade of inflammation, which results in irreversible neurodegeneration in various diseases (8, 9), including uveitis (10), glaucoma (11), age-related macular degeneration (AMD) (12), and retinitis pigmentosa (RP) (13). Thus, microglial activation plays an important role in a large number of inflammatory diseases.

Recently, interventions aimed at inhibiting the activation of microglia in neuroinflammatory disease have gained considerable interest. αB-Crystallin (CRYAB/HSPB5), a member of the small heat shock protein (sHSP) family, has been a recent target of interest for therapy. sHSP family members, induced by numerous stressors, are observed throughout the species (14). Recent studies have shown that αB-crystallin is not only an intracellular chaperone (stabilizing the correct protein conformation, folding, and translocation from multiple stresses) (15), but also acts as a signaling molecule in the extracellular space, communicating with other cells such as microglia, to regulate immune response and inflammation (16, 17). As an immunomodulatory neuroprotectant, αB-crystallin is involved in several stress-related statuses such as stroke (18), spinal cord contusion (19), and autoimmune demyelination (20). Furthermore, studies report that αB-crystallin alleviates neuroinflammation through inhibition of microglial activation in the anterior ischemic optic neuropathy (AION) (21) and experimental autoimmune encephalomyelitis (EAE) (17). Interestingly, retinal αB-crystallin is upregulated in *Staphylococcus aureus*-induced endophthalmitis and protects the retina from damage. Thus, modulation of the microglial activation by αB-crystallin in ocular inflammatory disease requires further investigation, which may lead to promising new therapy for uveitis.

Autophagy is a cellular process that eliminates aggregated or unfolded proteins to maintain protein homeostasis. It also removes excess or damaged organelles in the cells through several processes, including macroautophagy, microautophagy, and chaperone-mediated autophagy (CMA) (22, 23). In addition to maintaining natural innate proteostasis, molecular chaperones also participate in autophagy. As a form of molecular chaperone, αB-crystallin is closely linked with autophagy. Studies suggest that αB-crystallin not only modulates the autophagy of retinal pigment epithelium (RPE) in AMD (16) and the astrocyte in Parkinson's disease (24), but also the autophagy of cardiomyocytes in cardiomyopathy (25). Moreover, an increasing number of studies have shown that the upregulated levels of autophagy could promote microglial activation, leading to neuroinflammation, for example, in HIV associated encephalitis (26), intracerebral hemorrhage (ICH) (27), and cocaine exposure (23). In addition, one investigator reported that suppressing autophagy could inhibit microglial classical activation (28). Therefore, the role that αB-crystallin plays in microglial autophagy requires additional investigation; particularly, as microglial autophagy may provide a potential method of influencing microglial activation and ultimately ocular neuroinflammation.

Whether exogenous αB-crystallin can play a role in microglial autophagy and inhibit microglial activation in acute ocular inflammation remains unclear. Based on existing studies, we hypothesized that αB-crystallin may suppress microglial activation and influence autophagy to alleviate neuroinflammation. We aimed to explore the potential of αB-crystallin in the alleviation of ocular inflammatory diseases such as uveitis. For this purpose, the endotoxin-induced uveitis (EIU) mouse model, a mature model developed for acute ocular inflammation, was chosen. We divided our study into two parts, *in vitro* and *in vivo*. This study aimed to investigate the role of exogenous αB-crystallin in regulating microglial autophagy and activation in the BV2 microglia cells and the EIU mouse model.

## Materials and Methods

### Cell Culture and Treatment

BV2 cell lines (mouse microglial cell lines) were purchased from a commercial cell bank (DMSZ, Germany). The cells were cultured in Dulbecco's Modified Eagle Medium (DMEM) (C11330500BT, Gibco), containing 10% fetal bovine serum (FBS) (LB-10270, Roby), at 37°C in a humidified incubator with 5% CO_2_. BV2 microglial cells were randomly divided into three groups: (1) a control group, (2) a group treated with phosphate buffered saline (PBS)+ lipopolysaccharides (LPS), and (3) a group treated with αB-crystallin + LPS. The BV2 cells were seeded in six-well plates, followed by the addition of recombined human αB-crystallin (C7944-53, Us Biologycal) (1 μg/ml) or sterile PBS (C10010500BT, Gibco) 12 h before treatment with LPS. The selection for appropriate concentration of αB-crystallin was shown in the Supplementary Figure 1A. For LPS stimulation, LPS (L6529, Sigma) (100 ng/ml) was added into the six-well plates for the PBS+LPS or αB+LPS groups. The cells from one well were harvested at 6 h or 12 h after LPS stimulation for further analysis.

### EIU Mouse Model and Treatment

All animal studies were conducted in accordance with the Association for Research in Vision and Ophthalmology resolution and approved by the Zhongshan Ophthalmic Center Animal Care and Use Committee, Sun Yat-sen University, Guangzhou, China (authorization number 2019164). The C57BL/6J mice (6–8 weeks old) were maintained in a 12 h light/dark cycle at 23°C with *ad libitum* access to food and water. They were randomly divided into three groups: (1) a control group, (2) a PBS+LPS group, and (3) an αB+LPS group. The right eyes of mice in the PBS+LPS and αB+LPS groups were intravitreally injected with PBS and LPS (PBS+LPS group) or αB-crystallin and LPS (αB+LPS group). The EIU mouse model was induced by a single intravitreal injection of 1 μl LPS (200 μg/ ml). Twenty four hour before the LPS injection, mice in the PBS+LPS group received one intravitreal injection of 1 μl PBS. Mice in the αB+LPS group received one intravitreal injection of 1 μl αB-crystallin (500 μg/ml). For further analysis, mice in each group were sacrificed and the right eyeballs were enucleated at 6, 12, and 24 h after the LPS stimulation. The results for single intravitreous injection of αB-crystallin were shown in the Supplementary Figures 1B–D.

### Quantitative Real-Time Polymerase Chain Reaction (qRT-PCR)

Total RNA was isolated from the BV2 microglia of one well of six-well plates (*n* = 3) or the mouse retinas (*n* = 3) using RNAiso Plus (9108, Takara). RNA was subject to reverse transcription to cDNA using the PrimeScriptTMRT reagent kit (RR036B, Takara). The nucleic acid purity was quantified and analyzed using spectrophotometry (NanoDrop Technologies, Wilmington, DE). Primer sequences are presented in Table 1. Gene expression levels were measured using the LightCycler 480 system (Roche, Switzerland). The PCR procedure was as follows: pre-incubation for 5 min at 95°C, followed by 40 cycle amplification of denaturation for 10 s at 95°C, and annealing for 15 s at 60°C. The expression level of each gene was expressed as the fold expression after normalization to the internal control glyceraldehyde 3-phosphate dehydrogenase (GAPDH).

**Table 1:** Primer sequences for qRT-PCR.

**Gene**		**Primer sequence (3′~ 5′)**
IL6	Forward	TAGTCCTTCCTACCCCAATTTCC
	Reverse	TTGGTCCTTAGCCACTCCTTC
TNFα	Forward	TGCCTATGTCTCAGCCTCTT
	Reverse	GAGGCCATTTGGGAACTTCT
COX2	Forward	TCA TTC ACC AGA CAG ATT GCT
	Reverse	AAG CGT TTG CGG TAC TCA TT
iNOS	Forward	CCC TTC AAT GGT TGG TAC ATG G
	Reverse	ACA TTG ATC TCC GTG ACA GCC
Beclin1	Forward	GCACCATGCAGGTGAGCTTC
	Reverse	TTTCGCCTGGGCTGTGGTAA
GAPDH	Forward	GGTTGTCTCCTGCGACTTCA
	Reverse	TGGTCCAGGGTTTCTTACTCC

### Western Blotting

The total protein was extracted from the BV2 cells of one well of six-well plates (*n* = 3) or the mouse retinas (*n* = 3) using lysis buffer (KGP250, KeyGen) containing protease and phosphatase inhibitors. The protein concentration was measured using the PierceTM BCA Protein Assay Kit (23227, Thermo Scientific). An equal amount of protein from each sample was mixed with 5X SDS loading buffer (KGP101, KeyGen), subjected to 12% SDS gel, and then transferred to PVDF membranes (ISEQ00010, Merck Millipore). The membranes were blocked with 5% non-fat milk (MB3217, Meilunbio) in PBS for 1.5 h at room temperature and then incubated with primary antibodies against a nitric oxide synthase (iNOS) (ab15323, Abcam), cyclooxygenase 2 (COX2) (12282S, CST), Beclin1 (3738S, CST), Light Chain 3 (LC3) (3868S, CST), and GAPDH (ab8245, Abcam) overnight at 4°C. The membranes were then incubated with secondary antibodies (ab6802, Abcam; ab6789, Abcam) for 1 h. Protein bands were visualized using the ChemiDoc Touch Imaging System (Bio-Rad, USA). The band intensity was quantified using Image J software (NIH, USA).

### Enzyme-Linked Immunosorbent Assay (ELISA)

The cellular supernatant and intraocular liquid from mice were collected. Cardiac perfusion was performed with PBS on the anesthetized mice before they were sacrificed, followed by removal of the right eyes. The extraocular muscle and fascia were cleared. The eyeball was washed with PBS and dried with gauze. The eyeball was carefully dissected to remove the cornea, iris, lens, retina, and choroid; the intraocular liquid was then collected with tips. The cellular supernatant or intraocular fluid was collected and centrifuged at 16,000 *g* for 30 min at 4°C. The supernatant was removed and stored at −80°C until further analysis. The cell supernatant (*n* = 3) and intraocular fluid (*n* = 9) were collected 12 h after LPS stimulation. The cellular supernatant (1:50 diluted by PBS) and intraocular fluid (1:20 diluted by PBS) of each group were measured using ELISA kits for tumor necrosis factor alpha (TNF-α) (MTA00B, R&D) and interleukin-6 (IL-6) (M6000B, R&D), following the manufacturer's instructions.

### Immunofluorescent Staining

Cells were seeded onto 8-well glass chamber slides (PEZGS0816, Merck Millipore) for fixation and further staining. Cells were fixed with 4% paraformaldehyde (PFA) (DF0133-4, Leagene) for 15 min and washed by PBS three times. For cryosections, eyeballs were carefully removed, fixed in 4% PFA at 4°C overnight, subjected to gradient dehydration with sucrose solution (10, 20, and 30%), and embedded in an OCT compound (4583, Sakura) at −80°C. The frozen samples were sliced transversely (12 μm) with a cryostat (CM1950, Leica) at −20°C and then washed by PBS three times. For retinal flat mounts, the eyeballs were fixed in 4% PFA for 1 h at room temperature, and the retina was dissected out as a cup. The cells, cryosections, and retinal cups were blocked with PBS containing 0.1% Triton-X100 (1139ML100, Biosharp) and 5% bovine serum albumin (BSA) (4240GR100, Biofoxx) at room temperature for 1 h. The cells and cryosections were incubated with primary antibodies at 4°C overnight, and the retinal cups, for 24 h. The primary antibodies were diluted in blocking buffer. The primary antibody included anti-Iba1 (011-27991, Woka), anti-Beclin1 (ab207612, Abcam), and anti-LC3 (3868, CST). The reason we excluded P62 antibody was shown in the Supplementary Figure 1E. After washing with PBS three times, the cells, cryosections, and retinal cups were incubated with secondary antibodies for 2 h at room temperature. The secondary antibodies were conjugated with Alexa Fluor 488 (ab150129, Abcam) and Alexa Fluor 555 (A32794, Invitrogen). The cells and cryosections were counterstained with 4′,6-diamidino-2-phenylindole (DAPI) (C0065-50, Solarbio) for 5 min and washed by PBS three times. The retinal cups were cut into four pieces and flat-mounted with an anti-fade mounting medium (S2100, Solarbio). Images were captured using a confocal microscope (LSM880, Carl Zeiss). The immunofluorescent intensity was quantified using Image J software.

### Clinical Evaluation of Ocular Inflammation

After LPS injection for 24 h, the mice were anesthetized with an appropriate dose of phenobarbitone (50 mg/kg, intraperitoneal injection). The mice were administered tetracaine and tropicamide before the examination. The severity of the anterior and posterior segmental inflammation of the right eye was evaluated with slit lamp biomicroscopy (SL-D7/DC-3/IMAGEnet, Topcon) and the fundus imaging system (MicronIV, Phoenix). For the anterior segmental images, the eyeball was adjusted to a suitable position to observe and photograph the inflammation in the anterior segment. For the posterior segmental images, methylcellulose was applied to the ocular surface to maintain contact with the lens, and to acquire the fundus image. Tobramycin ointment was then applied to protect the cornea until palinesthesia. Images were captured for further analysis. The data were shown in the Supplementary Materials.

### Optical Coherence Tomographic (OCT) Imaging

Mice (*n* = 6) were anesthetized with pentobarbital (50 mg/kg, intraperitoneal injection), and their pupils were dilated with tropicamide. Retinal structure was assessed using an OCT imaging system (SpectralisOCT, Germany). The scan area centered on the optic nerve was 9 x 9 mm (496 data points/A scan, 1536 A-scans/horizontal B-scan, 85000 A scan/s, 30° × 30°, an average of three frames per B-scan). Saline was used to keep the cornea moist for the transparence of optical media to guarantee the image acquisition quality to be above 15. Retinal thicknesses (range of one diameter of optic disc distant from the margin of optic nerve head) were measured using the “measure” tool in the software. Total retinal thickness was measured from the nerve fiber layer to the RPE layer.

### Histopathological Analysis

The enucleated eyes (*n* = 6) were fixed in FAS solution (G1109, Guge) for 24 h, washed with PBS, dehydrated using gradient reagent alcohol (65, 75, 85, 95, and 100%), and then embedded in paraffin. Mice eye sections throughout the optic disc were cut at 5 μm thickness, deparaffinized, and stained with hematoxylin and eosin (H&E) (DH0006-2, Leagene) for histopathologic analysis. The number of intraocular inflammatory cells was calculated to assess the severity of uveitis symptoms. The sections throughout the optic disc were photographed using a microscope (Leica DM4000, Germany). Each image was captured with the optic disc as the center for counting inflammatory cells. A marquee of the same size (length: 6.5 times the scale, width: 5.5 times the scale) was applied across the optic disc and the number of inflammatory cells in it was counted. The counting was performed separately by two experienced researchers and the average of the numbers from each researcher were documented.

### Apoptosis by Terminal Deoxynucleotidyl Transferase dUTP Nick End Labeling (TUNEL) Assay

Cryosections were obtained using the before mentioned method. A TUNEL kit (12156792910, Roche) was used. The cryosections (*n* = 6) were washed by PBS three times and incubated the cryosections with 0.1% Triton-100 at 4°C for 5 min. The sections were incubated with the TUNEL reaction mixture at room temperature for 1 h, and then washed with PBS three times. The sections were then stained with DAPI for 5 min and washed three times. Finally, the sections were mounted with an anti-fade mounting medium and photographed using a confocal microscope (LSM880, Carl Zeiss). Images of the mid-peripheral retina were taken to count TUNEL positive cells in the visual field. The counting was performed by two researchers and the average numbers were documented.

### Visual Evoked Potential (VEP)

VEP recordings of mice (*n* = 6) were performed using an electrophysiological system (Diagnosys Celeris, USA). All experimental mice underwent a dark adaptation for 12 h prior to the daytime tests. Mice were anesthetized with pentobarbital (50 mg/kg, intraperitoneal injection). Pupils were dilated with tropicamide, and the cornea was anesthetized with tetracaine. VEP was recorded using a gold-plated wire loop electrode contacting the cornea as an active electrode. Stainless steel needles inserted under the skin at the middle part of the skull and the tail were the reference and ground electrodes, respectively. The amplitudes of the VEP wave were recorded as the average of three responses under 0.05 cd·s/m^2^ flash stimuli intensity. Examination parameters were as follows: LED intensity, 100%; frame rate, 6 ms; waveform contrast, 100%; and waveform frequency, 0.5 Hz.

### Transmission Electron Microscopy (TEM)

BV2 cells (*n* = 3) were collected from the six-well plates, pelleted by centrifugation and fixed with TEM fixative (G1102, Servicebio) at 4°C. Pellets were then washed with 0.1 M phosphate buffer (PB) (pH 7.4) three times before being pre-embedded in 1% agarose and fixed with 1% osmium tetroxide (OsO_4_) (Ted Pella Inc., USA) for 2 h at room temperature. Following fixation, samples were washed 3 times with PB and then dehydrated using an alcohol gradient (30, 70, 80, 95, and 100%) and two washes with 100% acetone (Sinopharm Chemical Reagent Co., China). To embed samples in resin, two different ratios of acetone to EMBed 812 (Structure Probe Inc., USA) were used; either 1:1 for 2 h or 1:2 overnight. Resin blocks were placed at 65°C for 48 h to polymerize. Blocks were sectioned into 60–80 nm slices using the EM UC7 ultramicrotome (Leica, Wetzlar, Germany). Slices were fixed onto cuprum grids (150 mesh) with formvar film and then stained with 2% uranium acetate and 2.6% lead citrate to avoid CO_2_ exposure. Grids were left to dry overnight at room temperature before being imaged using the HT7700 TEM system (Hitachi, Japan). Five fields were randomly selected for each sample and photographed. The number of autophagolysosomes in the field was recorded. Finally, the average number of the five fields was recorded as the result for the sample. Autophagolysosome is a single membrane hybrid structure generated by the fusion of autophagosome and lysosome, containing partially or fully degraded organelles as well as cytoplasmic material, and containing electron-dense regions representing undegraded residuals (29–33).

### Statistical Analyses

Statistical analyses were performed using SPSS 22.0 (IBM, USA). The mean ± SEM value comparisons of multiple groups were analyzed using one-way analysis of variance (ANOVA) followed by Tukey's *post hoc* test. A value of p < 0.05 was considered statistically significant.

## Results

### αB-Crystallin Inhibited the Release of Inflammatory Cytokines and the Activation of BV2 Cells

**Figure 1:**
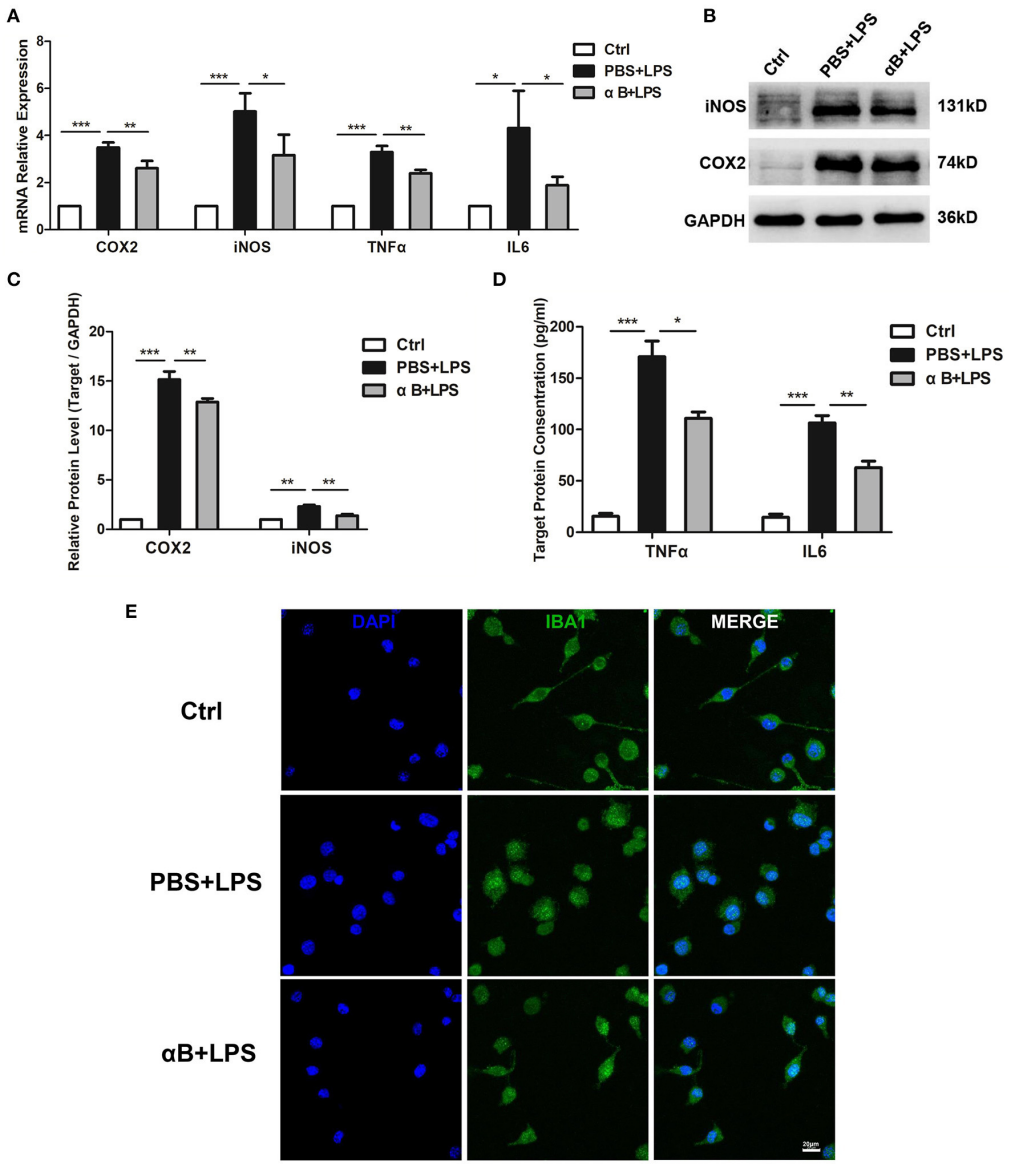
αB-Crystallin inhibits the release of inflammatory cytokine and the activation of microglia *in vitro*. **(A)** αB-Crystallin decreased the expression of COX2, iNOS, TNFα, and IL6, at the mRNA level, in BV2 cells exposed to LPS. **(B–D)** αB-Crystallin reduced the expression of inflammatory cytokines at the protein level caused by LPS. **(B,C)** Western bolt results for COX2 and iNOS. **(D)** ELISA results for TNFα and IL6. **(E)** αB-Crystallin inhibited the activation of BV2 cells by morphology. LPS induced BV2 cells to show an amoeboid appearance, while αB-crystallin suppressed this transformation. Scale bar 20 μm. *p < 0.05, **p < 0.01, and ***p < 0.001 (one-way ANOVA).

After the cultured BV2 microglia cell lines were stimulated by LPS for 6 and 12 h, the mRNA and the protein levels of inflammatory cytokines (including COX2, iNOS, TNFα, and IL6) were significantly upregulated when compared with those in the control group. Pre-treatment of cells with αB-crystallin reduced the expression of inflammatory cytokines at the mRNA and protein levels (Figures 1A–D). In terms of cell morphology, the control BV2 cells were flat with filopodia. When challenged with LPS, BV2 cells were activated, as represented by a round shape with retracted filopodia. Pre-treatment with αB-crystallin sustained the ramified microglial morphology (Figure 1E). In conclusion, αB-crystallin efficiently suppressed the activation of microglia and blocked the inflammation *in vitro*.

### αB-Crystallin Suppressed Autophagy of BV2 Cells

**Figure 2:**
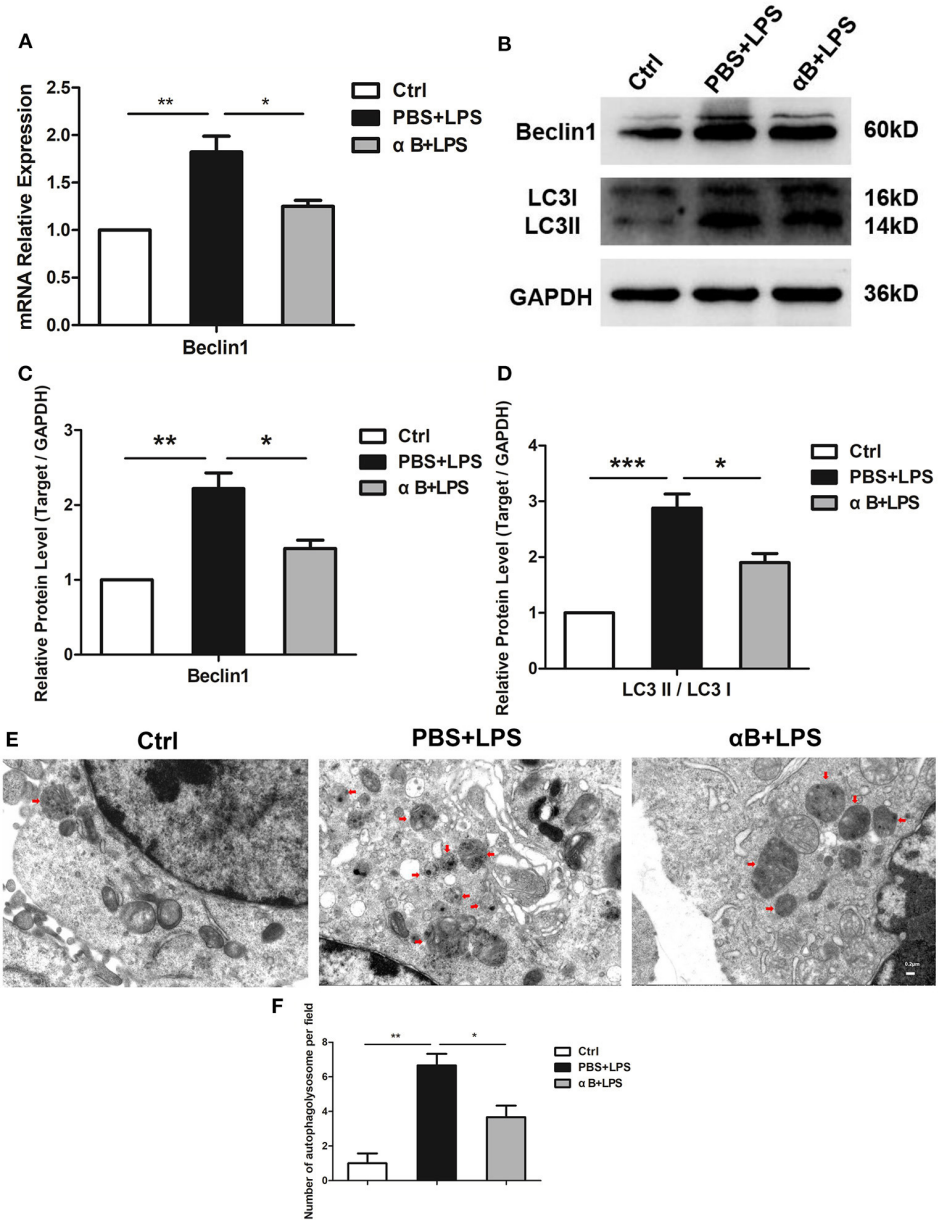
αB-Crystallin suppresses the autophagy of microglia *in vitro*. **(A)** LPS increased the mRNA expression of Beclin1(autophagy-related gene) in BV2 cells; αB-crystallin decreased its expression caused by LPS. **(B–D)** LPS increased the Beclin1 expression and the conversion of LC3I to LC3II at the protein level in BV2 cells. **(E)** The electron micrograph images and **(F)** the statistical results for the number of autophagolysosome showed that the rest of the BV2 microglia maintained low levels of autophagy. LPS induced obvious autophagy in BV2 cells. αB-Crystallin inhibited the autophagic level caused by LPS. Red arrows indicate autophagolysosomes in cells. Scale bar 0.2 μm. *p < 0.05, **p < 0.01, and ***p < 0.001 (one-way ANOVA).

Beclin1 and LC3II play an important role in the autophagy process. Beclin1 contributes to the maturation of the autophagosome, whereas LC3II is an indispensable molecule for the formation of the autophagosome membrane. After the LPS stimulation, the Beclin1 mRNA in BV2 increased at 6 h (Figure 2A); furthermore, a Beclin1 protein increase was observed at 12 h (Figures 2B,C). The transformation of LC3I to LC3II increased at 12 h at the protein level (Figures 2B,D). Autophagolysosomes (the end product of autophagy) were also observed, and LPS increased the number of autophagolysosomes in the cells (Figures 2E,F). Therefore, LPS led to an increase in autophagy. Nevertheless, pre-treating cells with αB-crystallin significantly inhibited the autophagy level in cells, as confirmed by the mRNA and protein levels and the intracellular ultrastructure (Figures 2A–F).

### αB-Crystallin Alleviated Inflammation of C57BL/6J Mice

**Figure 3:**
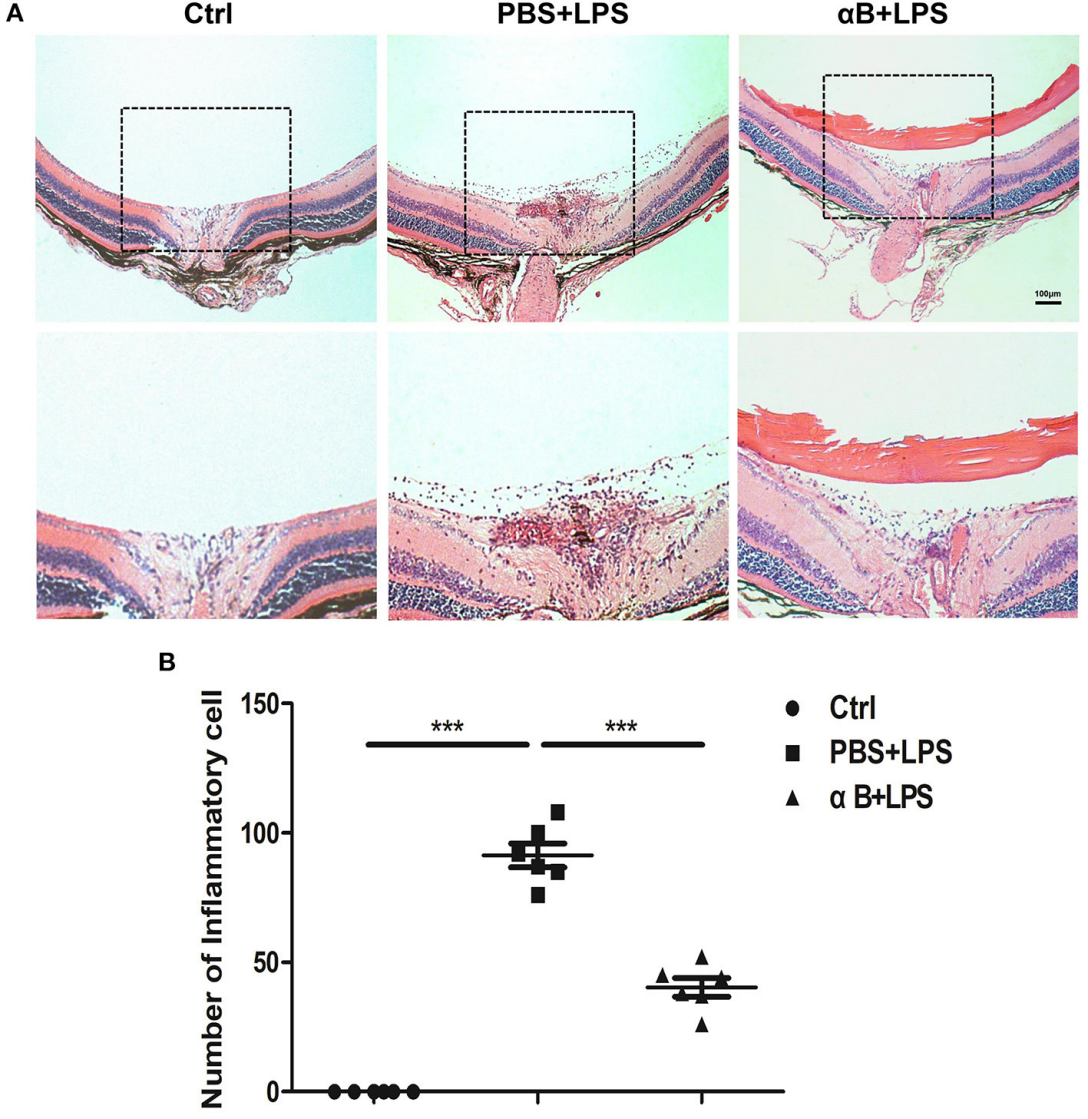
αB-Crystallin alleviates inflammation *in vivo*. **(A,B)** αB-Crystallin relieved inflammatory infiltration in C57BL/6J mice. **(A)** H&E staining images showed that αB-crystallin decreased the number of inflammatory infiltrating cells in the vitreous cavity of C57BL/6J mice caused by LPS. Scale bar 100 μm. **(B)** The scatter diagram of infiltrating cells. ***p < 0.001 (one-way ANOVA).

After the 24 h intraocular injection of LPS, we observed anterior and posterior segmental ocular inflammation. Anterior segmental images showed that LPS induced inflammatory reactions such as congestion, hypopyon, hyphema, and pupil synechia. Treatment with αB-crystallin suppressed the anterior ocular inflammation (Supplementary Figure 2A). Regarding the posterior inflammation, the image showed that LPS led to significant inflammation, including vitreous opacity, vascular white scabbard, optic disc edema, and inflammatory cell infiltration, whereas αB-crystallin inhibited these reactions (Supplementary Figure 2B). H&E pictures and the scatter diagram showed that αB-crystallin decreased the number of inflammatory cells infiltrating into the vitreous body caused by LPS (Figures 3A,B).

### αB-Crystallin Inhibited the Release of Inflammatory Cytokines and the Activation of Microglia in C57BL/6J Mice

**Figure 4:**
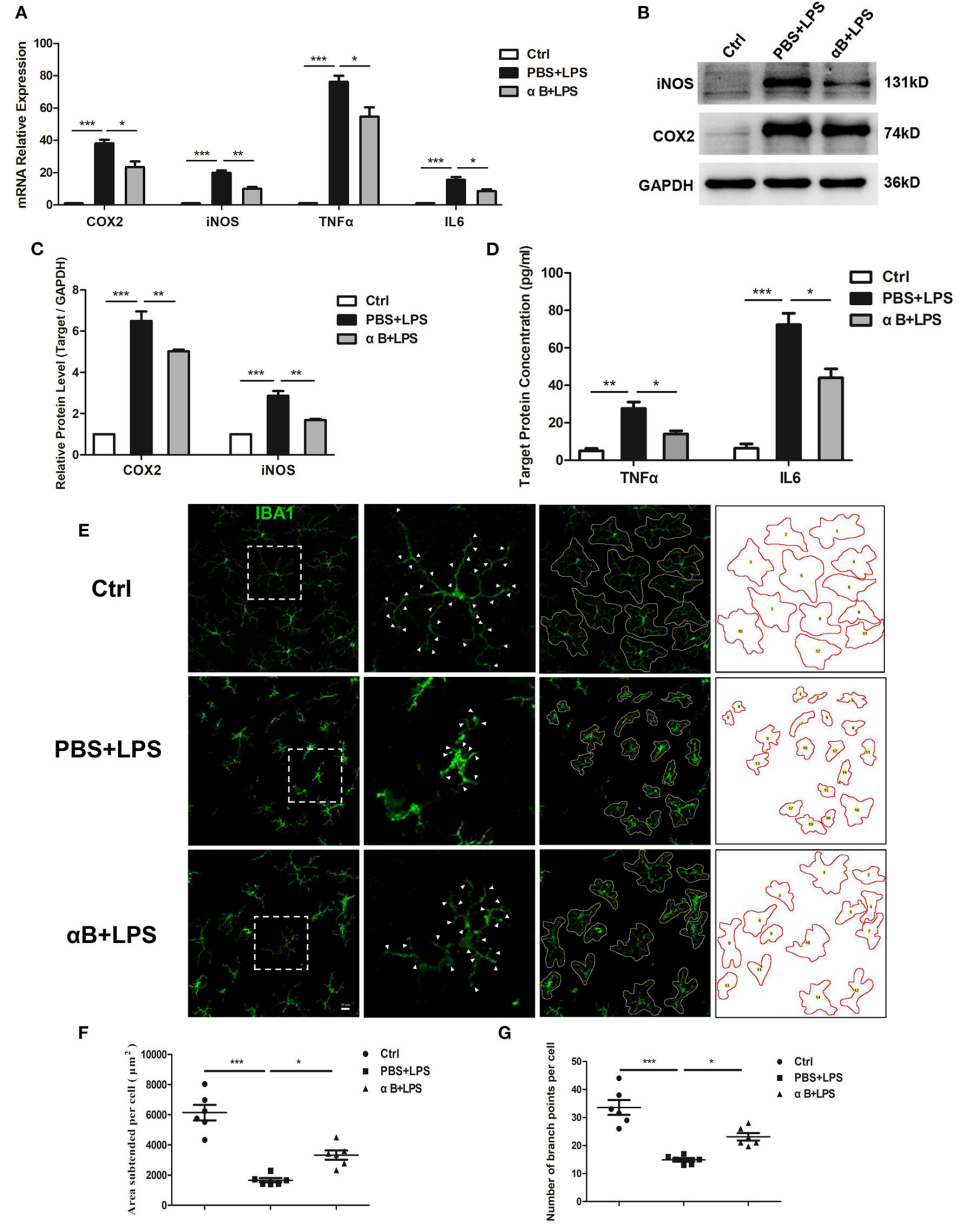
αB-Crystallin inhibits the release of inflammatory cytokine and the activation of microglia *in vivo*. **(A–D)** αB-Crystallin decreased the expression of COX2, iNOS, TNFα, and IL6, at mRNA level, in the retina exposed to LPS. **(B–D)** αB-Crystallin reduced the expression of these inflammatory cytokines at the protein level caused by LPS. **(B,C)** Western bolt results for COX2 and iNOS in the retina. **(D)** ELISA results for TNFα and IL6 in the intraocular fluid. **(E)** αB-Crystallin inhibited the activation of microglia in C57BL/6J mice caused by LPS. LPS changed the microglial morphology (less covering area and branches), whereas αB-crystallin suppressed this morphological transformation. Scale bar 20 μm. **(F)** The scatter diagram of area subtended per cell and **(G)** the number of branch points per cell. *p < 0.05, **p < 0.01, and ***p < 0.001 (one-way ANOVA).

The C57BL/6J mice were sacrificed, and the retina and vitreous fluid were tested for inflammatory cytokines (including COX2, iNOS, TNFα, and IL6). After LPS injection at 6 h and 12 h, the mRNA and protein levels of these inflammatory factors were significantly upregulated compared with those in the control group. Pre-treatment with αB-crystallin reduced the expression of inflammatory cytokines at both the mRNA and protein levels (Figures 4A–D). In terms of cellular morphology, retinal flat mounts showed that naive microglia presented a ramified shape with a large covering area and several branches. The activated microglia caused by LPS had an amoeboid shape with a small covering area and little branches. The ramified microglial morphology was sustained by αB-crystallin (Figure 4E). In addition to the direct morphologic changes, the statistical results of the subtended area and branches of each cell further confirmed the αB-crystallin suppression of microglial activation (Figures 4F,G).

### αB-Crystallin Suppressed Autophagy of Retinal Microglia in C57BL/6J Mice

**Figure 5:**
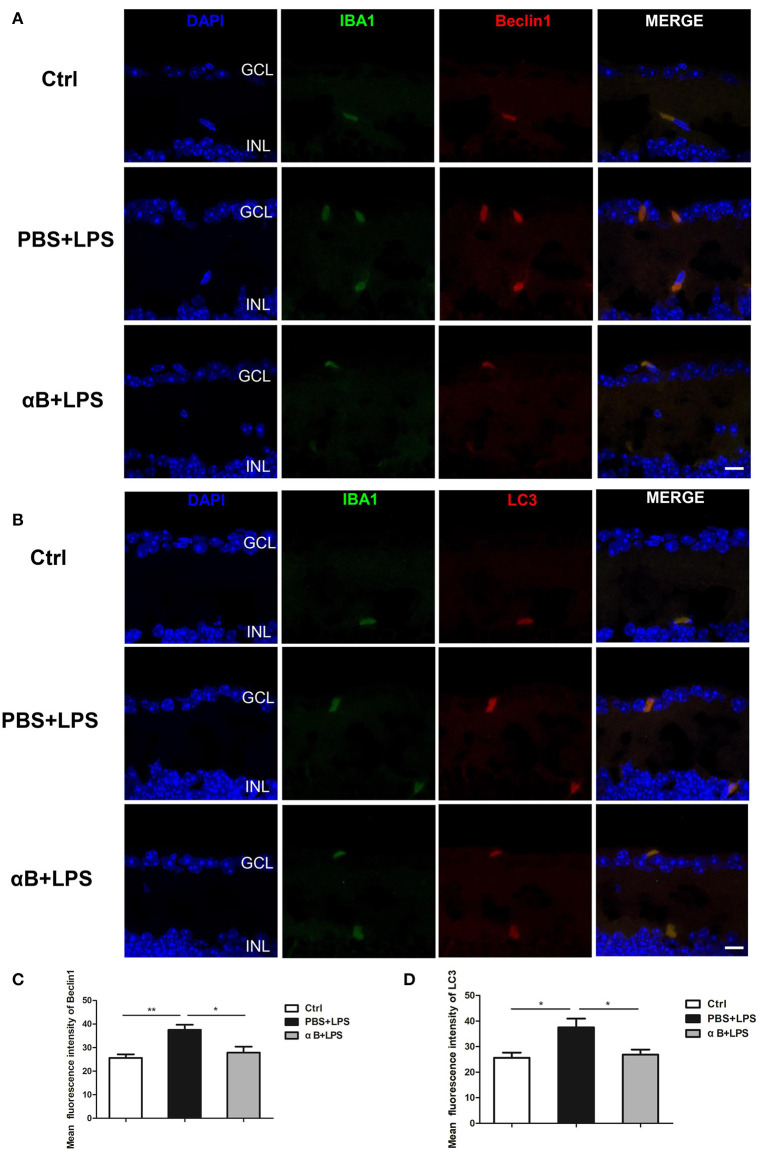
αB-Crystallin inhibits the autophagy of microglia *in vivo*. **(A)** Representative immunofluorescence images and **(C)** the histogram for mean fluorescence intensity of Beclin 1 showed that LPS increased the Beclin1 expression in microglia compared with that in the control group, and αB-crystallin inhibited the expression of Beclin1. **(B)** Representative immunofluorescence images and **(D)** the histogram for the mean fluorescence intensity of LC3 showed that LPS increased the LC3 expression in microglia, and αB-crystallin suppressed its expression. Scale bar 10 μm. GCL, ganglion cell layer. INL, inner nuclear layer. *p < 0.05 and **p < 0.01 (one-way ANOVA).

After the C57BL/6J mice were sacrificed, their eyeballs were collected for immunofluorescence assessment. The eyeball sections were double-stained for Iba1/Beclin1 or Iba1/LC3. The immunofluorescent images showed that Beclin1 and LC3 were mainly expressed in microglia, rather than other retinal cells, indicating that autophagy was an active biological process in the microglia cells. The results showed that LPS increased Beclin1 and LC3 expression compared with that in the control group, whereas αB-crystallin inhibited the expression of these autophagy-related proteins (Figures 5A–D).

### αB-Crystallin Contributed to Protecting the Structure of the Retina and Visual Function of C57BL/6J Mice

**Figure 6:**
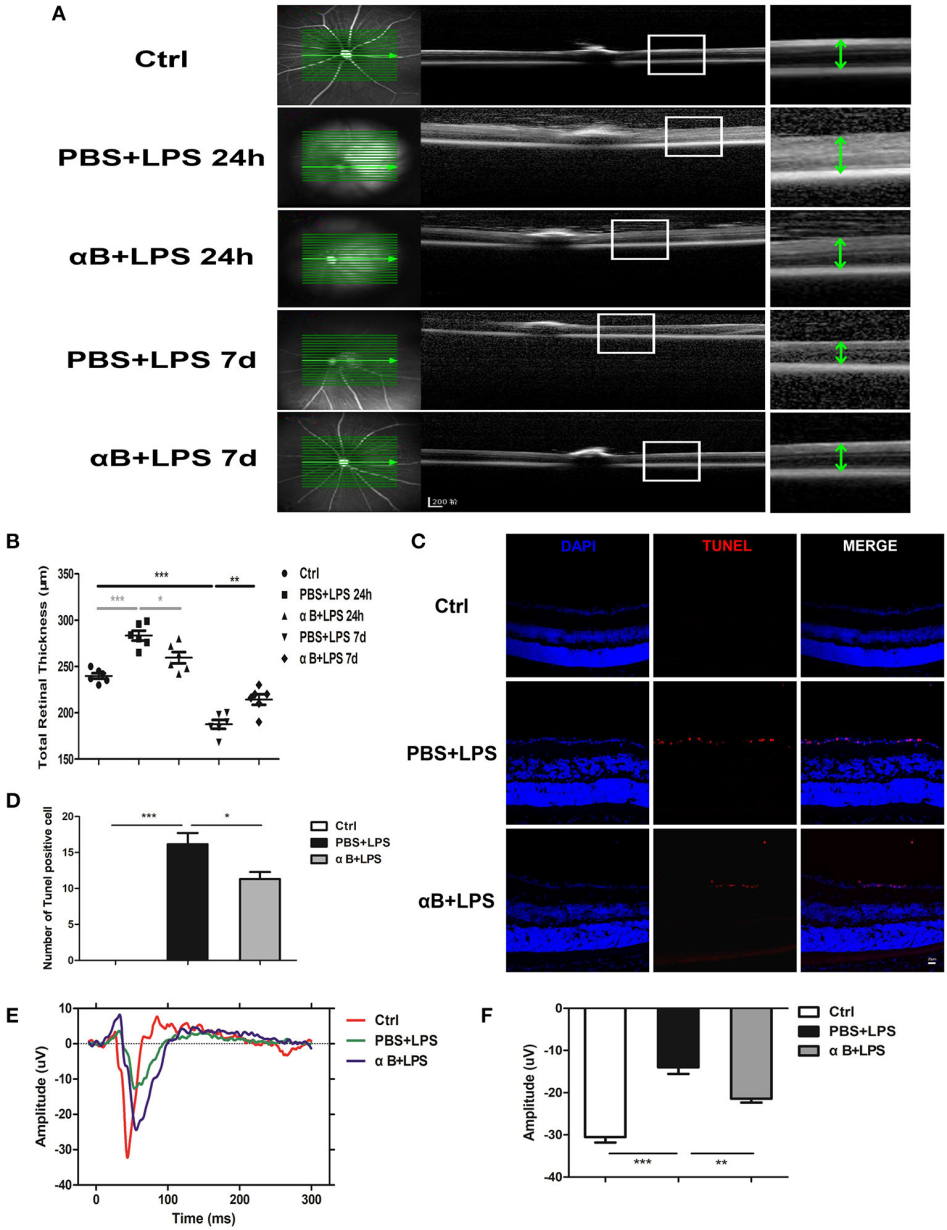
αB-Crystallin protects the structure of the retina and visual function of C57BL/6J mice. **(A,B)** Images of OCT **(A)** and scatter diagram **(B)** showed that retinal thickness increased at 24 h but decreased at 7 d in the PBS+LPS group, whereas αB-crystallin suppressed these changes. Scale bar 200 μm. **(C)** Representative immunofluorescence images and **(D)** the statistical results showed that LPS increased the TUNEL-positive cells in the retinal ganglion cell layer, whereas αB-crystallin decreased these cells. Scale bar 20 μm. **(E)** The waveform of VEP and **(F)** the statistical results showed that LPS decreased the amplitude of VEP, whereas αB-crystallin inhibited this decrease. OCT, optical coherence tomography. VEP, visual evoked potential. *p < 0.05, **p < 0.01, and ***p < 0.001 (one-way ANOVA). GCL, ganglion cell layer. INL, inner nuclear layer. ONL, outer nuclear layer.

Finally, we evaluated the protective effect of αB-crystallin on the mice retina. Intravitreal injection of LPS was performed 24 h later, and OCT was performed to detect the retinal thickness. LPS caused marked retinal thickening as well as increased retinal internal reflection indicating a severe retinal inflammatory reaction. Nevertheless, αB-crystallin decreased retinal thickness and retinal internal reflection. After 7 days, the thickness of the retina for the PBS+LPS group was reduced compared with that in the control group; thus, the inflammation might have caused further damage to the retinal structure. Regarding the LPS+αB-crystallin group, the retina was thicker than in the PBS+LPS group (Figures 6A,B). The TUNEL experiments showed that LPS induced retinal ganglion cell (RGC) death, whereas αB-crystallin reduced the TUNEL-positive cells (Figures 6C,D). In order to evaluate the function of the retina, we tested the VEP for mice at 7 d. Lower VEP amplitude showed that LPS induced functional impairment. This impairment was reduced by αB-crystallin, and the VEP amplitude for the αB+LPS group was higher than that for the PBS+LPS group (Figures 6E,F).

## Discussion

To determine the effect of αB-crystallin exposure on LPS-induced inflammation, microglial activation, and microglial autophagy, we utilized indicators representing inflammatory reaction, microglial activation, microglial autophagy, and retinal structure and function. We first examined whether αB-crystallin influences microglial activation and autophagy, and then investigated whether αB-crystallin can be used as an effective *in vivo* intervention for ocular inflammatory disease. We hypothesized that modulating microglial autophagy may provide a potential target for suppressing LPS-induced neuroinflammation.

Our results showed that prophylactic αB-crystallin treatment effectively reduced the inflammation and the activation of microglia *in vitro*. Similarly, Guo et al. (17) reported that αB-crystallin inhibited the expression of IL1β, IL6, and TNFα in microglia under stress conditions. Pangratz-Fuehrer et al. (21) concluded that αB-crystallin could dampen microglial activation. Holtman et al. (34) found that human microglia exposed to αB-crystallin induced a series of anti-inflammatory signals based on hub gene research. These results are consistent with the anti-inflammatory effects of αB-crystallin for sustaining cellular internal environment homeostasis. Differently, Bsibsi et al. (35) showed that microglia exposed to αB-crystallin (50 μg/ml) could induce IL6, TNF, and COX2 expression. Additionally, Bhat and Sharma (36) showed that α-crystallin could induce microglial activation. The likely reasons for this are as follows: the α-crystallin used was extracted from the bovine lens, whereas we used pure recombinant αB-crystallin. In their study (35, 36), they exposed microglia to α-crystallin (0–50 μg/ml) and found that a high concentration (50 μg/ml) of α-crystallin induced inflammation in microglia, whereas a low concentration of the protein did not. Thus, purity and protein concentration may influence the role of αB-crystallin in inflammation. In order to exert its protective effect, investigators need to ensure its purity and the appropriate concentration.

To the best of our knowledge, no reports currently exist for αB-crystallin in an EIU mouse model. In our study, we found that αB-crystallin reduced intraocular inflammation and inhibited the activation of microglia *in vivo*. Similarly, Holtman et al. (34) showed that systemic administration of αB-crystallin inhibited neuroinflammation. Arac et al. (18) demonstrated that αB-crystallin reduced both the stroke volume and the inflammatory cytokines. Wu et al. (37) reported that α-Crystallin inhibited microglial activation after the optic nerve crush. Despite the administration route (intravenous, intravitreal, or intraperitoneal) of exogenous αB-crystallin, the results show that αB-crystallin has a strong anti-inflammatory effect in various diseases, providing promising evidence for its use in alleviating ocular inflammatory diseases. This differs from the findings of Arac et al. (18) who showed that after stroke, the number of microglia cells was similar to the wild type. They only described the total number of microglia between the different groups and did not highlight the activated microglia of the CRYAB ^−/−^mice. Furthermore, Rao et al. (38) reported that αB-crystallin did not show a protective effect in experimental autoimmune uveitis (EAU). Their EAU mouse model was induced by subcutaneous injection of interphotoreceptor retinoid binding protein in B10.RIII mice, and the protective effect of crystallin was only evaluated on day 21 (EAU is a chronic process); this approach differed from ours. Studies suggest that the effect of α-crystallin might depend on the severity of oxidative stress and the duration of the stimulus (18). Moreover, results might differ between acute and chronic oxidative stress (39, 40). Therefore, the immunoregulatory role of αB-crystallin in different types of disease deserves further investigation.

The mechanism underlying αB-crystallin-mediated inhibition of microglial activation is still unclear (17). Studies show that increased autophagy might promote the activation of microglia and increase neuroinflammation in several instances, such as acute infection (41), acute electric stimulation (42), hypoxia (43), and traumatic brain injury (44). Yang et al. (45) found that suppressing autophagy decreased the microglial activation and inflammatory injury in ICH. Besides, François, et al. (46) found that through tri-culturing microglia, astrocytes, and neurons with LPS, only microglia exhibited an increased level of autophagy combined with upregulation of an inflammatory reaction. Therefore, autophagy might play an important role in the regulation of microglial activation and neuroinflammation (46). For this reason, we observed microglial autophagy in our study. Our results showed that αB-crystallin suppressed the microglial autophagy *in vitro* and *in vivo*. Similarly, Lu et al. (24) reported that CRYAB knockdown in astrocytes resulted in a marked augmentation of autophagy activity. In contrast, Kannan (16) concluded that extracellular αB-crystallin present in drusen increased the autophagy-mediated clearance. Pattison et al. (25) found that the mutation of αB-crystallin decreased the expression of Atg7 and reduced the autophagic function of rat cardiomyocytes. Therefore, αB-crystallin may either promote or inhibit cellular autophagy in different cells. To date, there have been several studies on autophagy, but no unanimous conclusion has been reached regarding its mechanism. Mizushima et al. (22) reported the apparent conundrum that autophagy had dual effects on cells, which might be either beneficial or detrimental. Numerous studies have shown that persistent, inefficient, or excessive induction of autophagy is detrimental and promotes cellular injury (47). Based on our results, αB-crystallin may prevent microglial immoderate autophagy, which may influence microglial activation (23). However, the specific relationship between microglial autophagy and microglial activation requires further investigation.

To our surprise, our results showed that autophagic markers were detected almost exclusively in microglia (in the retina) (Figures 5A,B). Therefore, autophagy played an active and vital role in the microglial biological process. Further studies are required to understand the effect of αB-crystallin on microglial autophagy, such as the possible pathway. It has been reported in the literature that αB-crystallin is the ligand for TLR2 (35, 48), and based on our results it can inhibit microglial autophagy induced by LPS (ligand for TLR4). Furthermore, both TLR2 and TLR4 participate in microglial autophagy (41, 45). Whether there is a connection between the two receptors during autophagy is unclear. A deeper understanding of αB-crystallin combined with preliminary clinical application provides a potential therapy not only for uveitis but also for other inflammatory diseases.

In summary, the present study showed that prophylactic αB-crystallin treatment can suppress the LPS-induced inflammation. It reduced the release of proinflammatory cytokines as well as the activation of microglia, both *in vitro* and *in vivo*. Simultaneously, we found that αB-crystallin inhibits microglial autophagy. Microglial autophagy may therefore play a role in microglial activation. Finally, we demonstrate its beneficial effects for protecting the structure and function of the retina in the EIU model. Taken together, the use of αB-crystallin could be considered a novel potential interventional strategy for acute ocular inflammatory diseases induced by microglial activation. Additionally, the regulation of microglial autophagy may be a new effective target for the alleviation of inflammatory diseases.

## Data Availability Statement

The original contributions presented in the study are included in the article/Supplementary Material, further inquiries can be directed to the corresponding author/s.

## Ethics Statement

The animal study was reviewed and approved by Zhongshan Ophthalmic Center Animal Care and Use Committee, Sun Yat-sen University, Guangzhou, China.

## Author Contributions

FW and ZJ performed experiments, analyzed data, and wrote the manuscript. XL designed and supervised the experiments. YY supervised the experiments and revised the manuscript. BL, FD, SQ, ZC, and XM performed part of the experiments. All authors contributed to the article and approved the submitted version.

## Conflict of Interest

The authors declare that the research was conducted in the absence of any commercial or financial relationships that could be construed as a potential conflict of interest.
