# Supplementary material for: αB-Crystallin Alleviates Endotoxin-Induced Retinal Inflammation and Inhibits Microglial Activation and Autophagy
Source: Front Immunol. 2021 Mar 11;12:641999. doi: 10.3389/fimmu.2021.641999 (PMC7991093; doi:10.3389/fimmu.2021.641999)
Supplement: Supplementary file 1 [file Data_Sheet_1.docx]

**
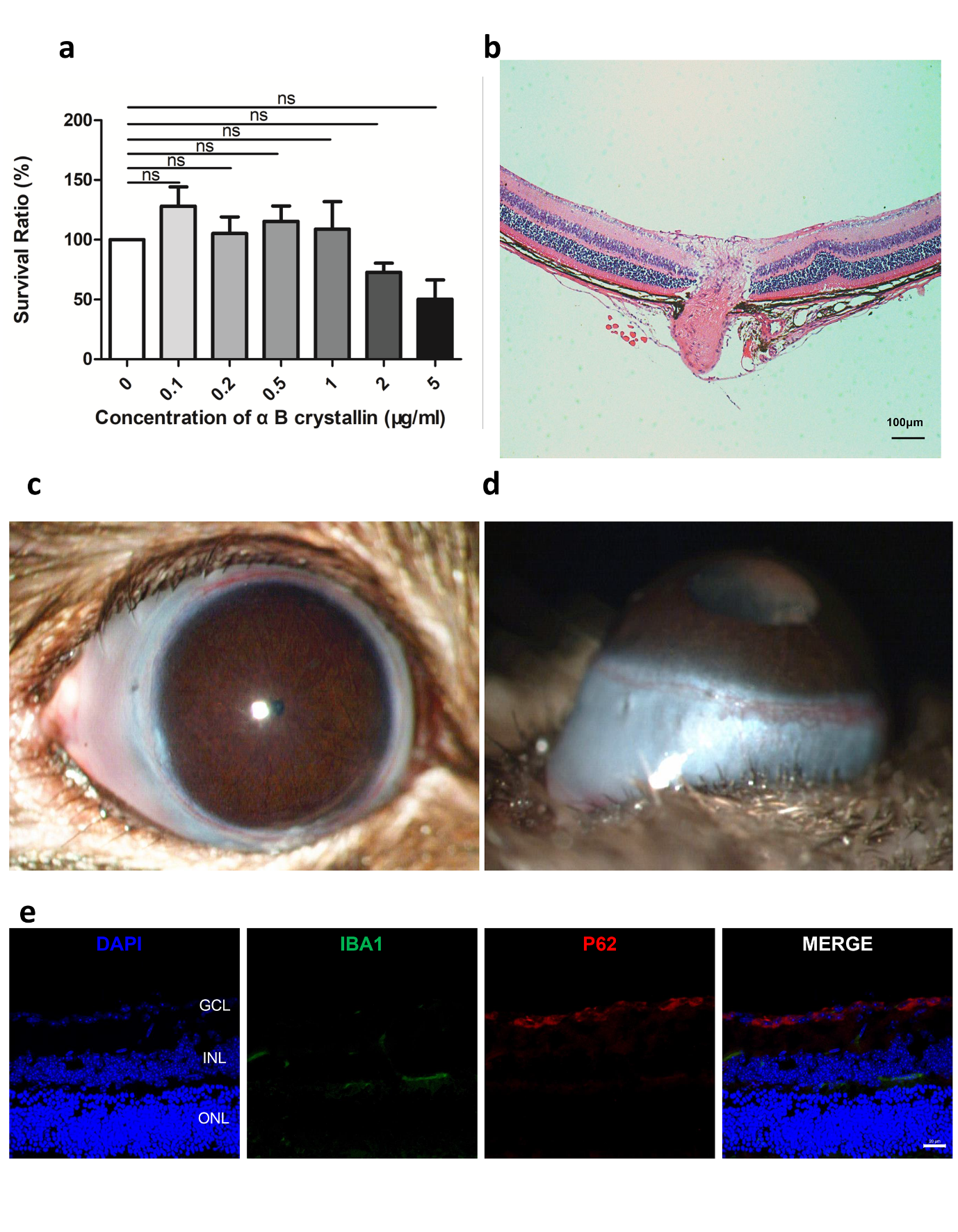
Supplementary materials**

**E**

**D**

**C**

**B**

**A**

**Supplementary Figure1. (A)** CCK8 results showed that different concentrations of αB-crystallin did not affect the survival rate of cells; however, injection of 2 μg/ml of αB-crystallin slightly decreased the survival rate of cells; the 1 μg/ml concentration was therefore chosen to be the best concentration. **(B-D)** Single-injection of αB-crystallin did not cause any observable inflammatory changes in the anterior or posterior segment. **(B)** HE-stained images showed that sporadic inflammatory cells exist after a single αB-crystallin injection (Scale bar, 100 μm) **(C, D)** Anterior segment photography showed only slight congestion after the αB-crystallin injection. **(E)** Control mice eyeball sections were double-stained for Iba1 and P62. P62 was primarily expressed in the retinal ganglion cell layer and did not coincide with green fluorescent-Iba1. We did not choose P62 as the autophagic indicator (Scale bar, 20 μm); therefore, P62 is not the indicator of microglial autophagy.


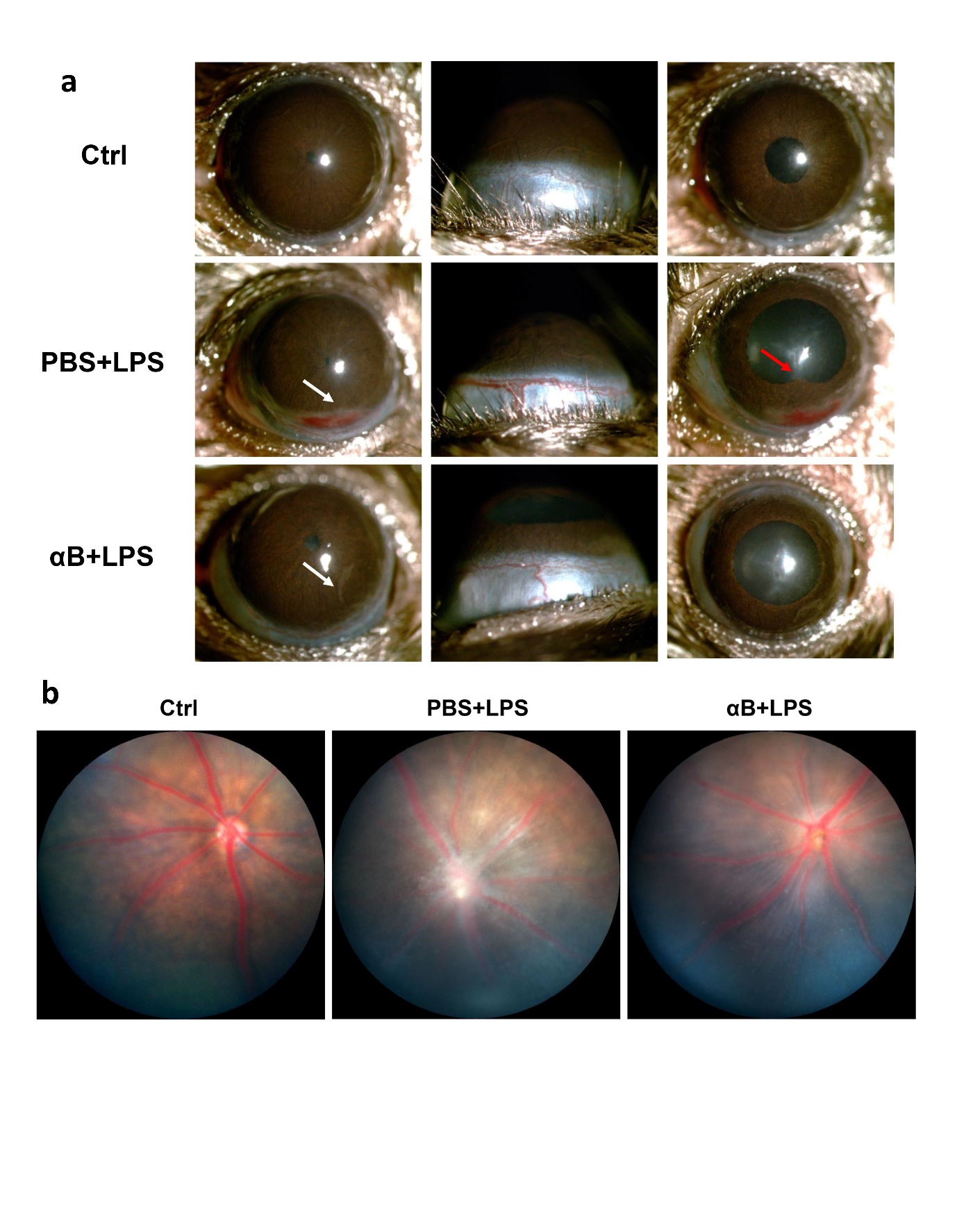


**B**

**A**

**Supplementary Figure2. (A, B)** αB-Crystallin relieved clinical features in C57BL/6J mice. **(A)** αB-Crystallin decreased inflammation in the anterior chamber caused by LPS (including congestion, hypopyon, hyphema, and pupil synechia). The white arrow indicates hypopyon. The red arrow indicates pupil synechia. **(B)** αB-Crystallin suppressed inflammation in the posterior segment caused by LPS (including vitreous opacity, vascular white scabbard, optic disc edema, and inflammatory cell infiltration).
